# Elevated serum levels of bone sialoprotein (BSP) predict long-term mortality in patients with pancreatic adenocarcinoma

**DOI:** 10.1038/s41598-018-38352-2

**Published:** 2019-02-06

**Authors:** Sven H. Loosen, Pia Hoening, Niklas Puethe, Mark Luedde, Martina Spehlmann, Tom F. Ulmer, David V. Cardenas, Sanchari Roy, Frank Tacke, Christian Trautwein, Ulf P. Neumann, Tom Luedde, Christoph Roderburg

**Affiliations:** 1 Department of Medicine III, University Hospital RWTH Aachen, Pauwelsstrasse 30, 52074 Aachen, Germany; 2 Division of Gastroenterology, Hepatology and Hepatobiliary Oncology, University Hospital RWTH Aachen, Pauwelsstrasse 30, 52074 Aachen, Germany; 3 Department of Internal Medicine III, University Hospital of Schleswig Holstein, Campus Kiel, Rosalind-Franklin-Str. 12, 24105 Kiel, Germany; 4 Department of Visceral and Transplantation Surgery, University Hospital RWTH Aachen, Pauwelsstrasse 30, 52074 Aachen, Germany

## Abstract

Patients with pancreatic adenocarcinoma (PDAC) still face a very limited prognosis. At early stage, surgical tumor resection might offer long-term survival but disease recurrence is common and the existing stratification algorithms are often unsuitable to identify patients who particularly benefit from surgery. Here, we investigated the potential role of bone sialoprotein (BSP) as a circulating marker in patients undergoing resection of PDAC. We used ELISA to determine serum concentrations of BSP in a cohort of 132 PDAC patients as well as 39 healthy controls. Circulating BSP levels were significantly higher in PDAC patients compared to healthy controls. Notably, elevated preoperative BSP levels above the ideal cut-off value of 4743 pg/ml turned out as a significant predictor for an impaired postoperative survival. The potential of preoperative BSP levels as a prognostic marker was further underlined by uni- and multivariate Cox-regression analyses including various tumour- and patient-specific. Finally, high tumoral BSP expression was also associated with a significantly impaired long-term survival. In conclusion, we identified a novel role of circulating BSP as a biomarker in PDAC patients undergoing tumor resection. Such data might help to establish new preoperative stratification strategies to better identify patients who particularly benefit from tumor resection.

## Introduction

Pancreatic adenocarcinoma (PDAC) is among the most deadly malignancies. Despite being responsible for only 3% of all new cancer diagnoses, pancreatic cancer represents the fourth most common cause of cancer related death in Europe^1,2^ and is expected to be the second most common cause by 2030^1^. Current treatment options are limited, with surgical resection being the only potentially curative treatment option^3^. However, most patients are diagnosed at advanced tumor stage and long-term survival cannot be achieved in these patients^2^. Moreover, even after radical tumor resection, some patients face early tumor recurrence and most likely do not benefit from surgery^4,5^. Thus, in order to improve long-term outcomes for pancreatic cancer patients, it is imperative to improve the proportion of patients diagnosed at an early disease stage and to identify those patients that will particularly benefit from radical treatment modalities, highlighting the need for easy accessible biomarkers for diagnosis and therapeutic guidance of pancreatic cancer patients^6^.

Pancreatic cancer is associated with a desmoplastic stroma reaction, which is critical for tumor progression and metastasis^7,8^. The stroma and the tumor itself express various proteins, which have been proven to be prognostic biomarkers^9,10^. In this context, “small integrin binding ligand N-linked glycoproteins” (SIBLINGs) have gained increasing interest due to their specific role in the regulation of tumor cell proliferation, angiogenesis and metastasis as well as their involvement in molecular processes in pancreatic cancer^11^. The SIBLINGs-family represents a class of soluble, integrin-binding glycophosphoproteins that includes bone sialoprotein (BSP), dentin sialophosphoprotein (DSPP), osteopontin (OPN), matrix extracellular phosphoglycoprotein (MEPE) and dentin matrix protein 1 (DMP1)^11^. SIBLINGs act on various receptors which are associated with different signalling pathways implicated in cancer^12^. Osteopontin represents the most investigated SIBLINGs-family member and was described to be strongly overexpressed in pancreatic cancer^13^. Besides osteopontin, BSP was recently found to be expressed in pancreatic islet and ductal cells of normal pancreatic tissues as well as in the tubular complexes of pancreatic cancer and pancreatic cancer cell lines, suggesting a role of BSP in the context of this malignancy^14^. With respect to circulating levels of the SIBLINGs-family, elevated serum levels of osteopontin were found in PDAC patients and correlated with disease stages and an impaired patients’ prognosis^15,16^. However, it is presently unknown if circulating levels of BSP play a similar role as a diagnostic and/or prognostic biomarker in PDAC patients. Here, we measured BSP serum levels in 132 PDAC patients at different stage of disease that underwent surgical tumor resection at our tertiary referral centre.

## Patients and Methods

### Patient characteristics and study design

The aim of this observational cohort study was to evaluate BSP as a biomarker in PDAC patients. A total of 132 patients who underwent resection of pancreatic adenocarcinoma at the Department of Visceral and Transplantation Surgery at the University Hospital RWTH Aachen were recruited between 2011 and 2016 (patient characteristics are summarized in Table 1). Diagnosis of PDAC was performed based on the patients’ medical history, physical examinations (silent jaundice, weight loss), imaging techniques (CT, MRI), laboratory analyses (elevated Bilirubin, GGT, AST, ALT, AP, and CA 19-9 concentration) as well as tumor biopsy and. All PDAC tumours were confirmed histopathologically after resection. Blood samples were taken prior to surgery and 6–7 days after tumour resection. We analysed 39 healthy, cancer-free blood donors with normal values for blood counts, C-reactive protein, kidney and liver function as control population. The protocol of the study was approved by the ethics committee of the University Hospital Aachen (RWTH Aachen University, Aachen, Germany) and the study was conducted in accordance with the ethical standards of the Declaration of Helsinki. We obtained written informed consent from all patients.

**Table 1:** Characteristics of study population.

Patients with PDAC undergoing surgical resection	132
Sex [%]:	
male-female	63.6–36.4
Age [years, median and range]	68.0 [41–84]
BMI [kg/m^2^, median and range]	24.53 [16.24–43.21]
PDAC characteristics [%]:	
T1-T2-T3-T4	3.7-3.7-86.9-5.6
N0-N1	29.9–70.1
M0-M1	82.9–17.1
G2-G3	52.0–48.0
R0-R1	68.4–31.6
Clinical performance status [%]:	
ECOG 0-1-2-3	53.0-33.3-9.4-4.3
Deceased during follow-up [%]:	
Yes-No	72.7–27.3

### Measurement of BSP serum levels

BSP serum concentrations were measured using a commercially available enzyme-linked immunosorbent assay (ELISA) in accordance to the manufacturers’ instructions (Human BSP ELISA, Abbexxa, Cambridge, United Kingdome, No. abx575181). The assay detects the un-phosphorylated form of human BSP of approximately 35 kDa. The specific BSP detection-antibody targets the immunogenic region between amino acids 17 to 210 from BSP.

### TGCA-PAAD

We downloaded the raw BSP mRNA data set from the TCGA-PAAD online portal (https://portal.gdc.cancer.gov/projects/TCGA-PAAD). The dataset comprised 178 samples of pancreatic cancer. The clinical data (e.g. survival time) of the resected patients were also taken from the TCGA data portal cBioPortal (http://www.cbioportal.org/index.do). The optimal prognostic cut-off value was determined as described previously^17^ and used to distinguish between patients’ survival on ‘low’ or ‘high’ BSP mRNA expression (threshold: −3.49 relative BSP mRNA expression level).

### Statistical analysis

Statistical analyses were performed as described recently^18^. Serum data are displayed as median and range. Shapiro-Wilk-Test was used to test for normal distribution. Non-parametric data were compared using the Mann-Whitney-U-Test or Kruskal-Wallis-Test for multiple comparisons. Box plots display a summary of the median, quartiles and ranges. ROC curves were created by plotting sensitivity against 1-specificity. The optimal cut-off values for ROC curves were established using the Youden-Index (YI = sensitivity + specificity − 1). Kaplan-Meier curves were used to display the impact on the patients’ survival. The Log-rank test was used to test for differences between subgroups in Kaplan-Meier curve analysis. The ideal prognostic cut-off value was calculated with a recently published biometric software that fits Cox proportional hazard models to the survival status (deceased during follow-up or alive) and the survival time. The ideal prognostic cut-off value is then defined as the value with the most significant split in the log-rank test^17^. The prognostic value of variables was further tested by uni- and multivariate Cox regression analysis. Parameters with a p-value of <0.250 in univariate testing were included into multivariate testing. Statistical analyses were performed using SPSS 23 (SPSS, Chicago, IL, USA)^19^. A p-value of <0.05 was considered statistically significant (*p < 0.05; **p < 0.01; ***p < 0.001).

## Results

### Circulating levels of BSP are elevated in patients with pancreatic cancer

**Figure 1:**
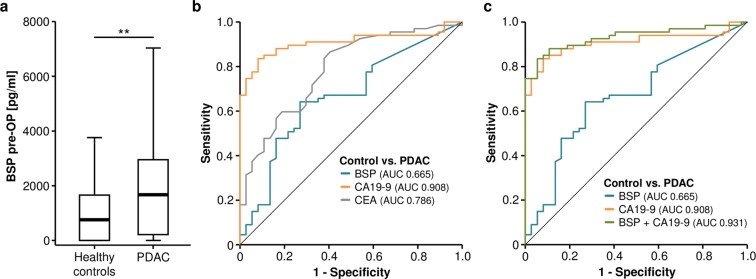
Circulating levels of BSP are elevated in pancreatic cancer patients. (**a**) Pancreatic cancer patients show significantly higher serum BSP levels compared to healthy controls. (**b**) Pre-operative BSP levels display an AUC value of 0.665 for the discrimination of pancreatic cancer patients and healthy controls. (**c**) The combination of CA19-9 and serum BSP levels shows the highest diagnostic power.

Based on recent data on an overexpression of BSP in pancreatic cancer samples^14^, we first measured preoperative circulating levels of BSP in a large cohort of patients (n = 132) who underwent tumor resection for pancreatic cancer (detailed patient characteristics are given in Table 1) and compared them to healthy controls. Interestingly, we found that pancreatic cancer patients display a significant elevation of BSP serum levels compared to healthy controls (Fig. 1a and Supplementary Table 1). Although the diagnostic power of serum BSP levels (AUC_BSP_: 0.665) for the differentiation of pancreatic cancer patients and healthy controls was inferior to tumor markers such as CA19-9 and CEA (AUC_CA19-9_: 0.908, AUC_CEA_: 0.786, Fig. 1b), the combinational use of BSP and CA19-9 was superior to each serum marker alone (AUC_BSP/CA19-9_: 0.931, Fig. 1c). The combination of BSP and CA19-9 displayed a maximum diagnostic sensitivity of 83.6% with a specificity of 94.6%.

### Circulating BSP does not reflect disease characteristics

In a next step, we compared serum BSP levels between pancreatic cancer patients with different clinicopathological disease characteristics such as TNM stage, resection status, histological tumor grading or the patients’ clinical performance status. In these analyses, we observed no significant difference in BSP serum levels between patients with different T-stage (Supplementary Fig. 1a), nodal-positive and nodal-negative disease (Supplementary Fig. 1b) or non-metastasized and metastasized patients, who were still eligible for surgical tumor resection (Supplementary Fig. 1c). Moreover, preoperative BSP serum levels had no impact on the resection status (R_0_ vs. R_1_, Supplementary Fig. 1d), while patients with poorly differentiated tumours (G3) showed a trend towards higher BSP level compared to patients with moderately differentiated tumours (G2, Supplementary Fig. 1e). Finally, serum BSP levels were unaltered between patients with an unimpaired or impaired clinical performance status (ECOG PS 0 vs. ≥1, Supplementary Fig. 1f). To further elaborate a potential association between circulating BSP and organ dysfunction, we performed correlation analyses between initial BSP levels and various laboratory markers. In this analysis, we observed a significant correlation between BSP serum levels and parameters of inflammation (CRP: R: 0.200, p = 0.033) as well as liver function (AST: R: 0.184, p = 0.038; bilirubin: R: 0.183, p = 0.039 and ALP: 0.190, p = 0.042, Supplementary Tables 1 and 2).

### Preoperative serum BSP levels predict long-term survival after tumor resection

**Figure 2:**
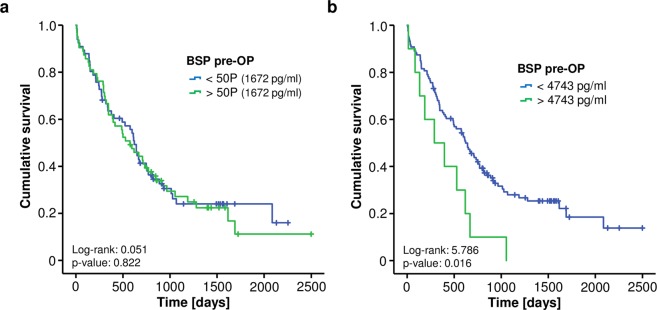
High preoperative serum levels of BSP are associated with a reduced overall survival after resection of pancreatic cancer. (**a**) The 50^th^ percentile of initial BSP serum levels is unsuitable to distinguish between patients with a favourable or unfavourable long-term prognosis. (**b**) Pancreatic cancer patients with preoperative BSP serum levels above the calculated ideal cut-off value (4743 pg/ml) have a significantly reduced overall survival of 289 days compared to 622 days for patients with BSP serum levels below this cut-off.

We next evaluated a potential association between preoperative BSP serum levels and the patients’ postoperative outcome. We therefore split our cohort into two groups with either high (above the 50^th^ percentile) or low (below the 50^th^ percentile) circulating levels of BSP and performed Kaplan-Meier curve analysis to test for significant differences in the overall survival between these groups. However, using the median BSP serum level (1672 pg/ml), we did not observe an impaired outcome for either group (Fig. 2a). In view of the fact that the median BSP level might not represent the optimal discriminator for patients with a good or poor prognosis, we subsequently calculated an ideal prognostic BSP cut-off value for the discrimination between long-term survivors and patients who deceased early after surgery as described before (see Patients and Methods for details)^17,18^. Interestingly, when applying this optimal cut-off value, Kaplan-Meier curve analysis showed a significantly impaired long-term survival for patients with preoperative BSP serum levels above 4743 pg/ml (Fig. 2b). Pancreatic cancer patients with BSP serum levels above 4743 pg/ml (n = 10) displayed a reduced median overall survival of only 289 days compared to 622 days for patients with preoperative BSP levels below the ideal cut-off value (n = 119). Importantly, none of the patients with high levels of circulating BSP reached long-term survival beyond three years (Fig. 2b).

Next, we performed univariate Cox-regression analysis to further elaborate the influence of high BSP levels on the patients’ survival. In this analysis, serum BSP levels above our ideal cut-off value were found to be a negative prognostic factor for overall survival showing a hazard ratio (HR) of 2.281 (p = 0.015, 95% CI: 1.117–4.420). In multivariate Cox-regression analysis, in which we included various clinical as well as laboratory parameters with a p-value of <0.250 in univariate testing, the prognostic value of circulating BSP was independent of the patients’ liver function (bilirubin and GGT), renal function (creatinine), tumor stage or patients’ age (Table 2).

**Table 2:** Uni- and multivariate Cox-regression analyses for the prediction of overall survival.

Parameter	Univariate Cox-regression		Multivariate Cox-regression	
	p-value	Hazard-Ratio (95% CI)	p-value	Hazard-Ratio (95% CI)
BSP (>4743 pg/ml)	0.015	2.281 [1.117–4.420]	0.029	2.199 [1.084–4.461]
Leukocyte count	0.710	0.987 [0.922–1.057]		
CRP	0.302	1.003 [0.998–1.008]		
Platelets	0.939	1.000 [0.998–1.002]		
AST	0.335	1.001 [0.999–1.004]		
ALT	0.726	1.000 [0.999–1.002]		
Bilirubin	0.074	1.039 [0.996–1.083]	0.359	1.025 [0.972–1.081]
ALP	0.549	1.000 [0.999–1.001]		
GGT	0.246	1.000 [1.000–1.001]	0.446	1.000 [1.000–1.001]
LDH	0.987	1.000 [0.988–1.012]		
Creatinine	0.013	1.014 [1.003–1.023]	0.758	1.084 [0.648–1.813]
BMI	0.950	1.001 [0.958–1.047]		
Age	0.003	1.031 [1.010–1.053]	0.054	1.027 [1.009–1.054]
Sex	0.893	0.972 [0.639–1.477]		
T-stage (T1/T2 vs. T3/T4) (T1/T2 vs. T3/T4)	0.060	3.046 [0.956–9.073]	0.061	3.108 [0.949–10.182]

BSP: bone sialoprotein, CRP: C-reactive protein, AST: aspartate transaminase, ALT: alanine transaminase, ALP: alkaline phosphatase, GGT: γ-glutamyltransferase, LDH: lactate dehydrogenase, BMI: Body-Mass-Index.

### Postoperative BSP serum levels and patients’ outcome

**Figure 3:**
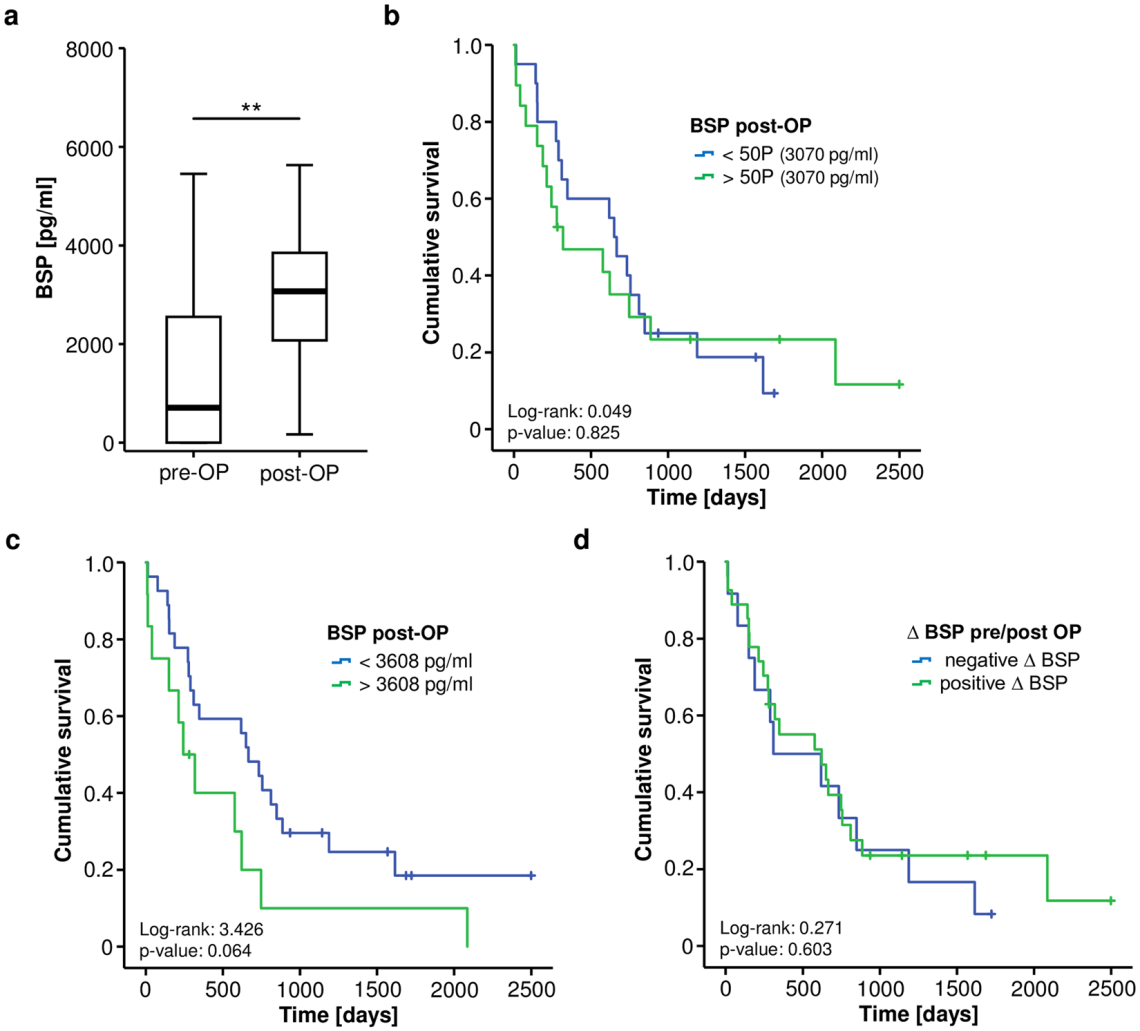
Postoperative BSP serum levels and patients’ outcome. (**a**) Postoperative BSP serum levels are significantly higher compared to the respective preoperative values. (**b**) The 50^th^ percentile of postoperative BSP serum levels is unsuitable to distinguish between patients with a favourable or unfavourable long-term prognosis. (**c**) Pancreatic cancer patients with postoperative BSP serum levels above the ideal cut-off value (3608 pg/ml) show a trend towards an impaired prognosis. (**d**) Longitudinal changes of BSP serum levels before and after surgery do not predict patients’ prognosis.

Subsequently, we measured BSP levels in postoperative serum samples that were drawn six to seven days after resection of pancreatic cancer and were available for n = 39 patients. Interestingly, we found that median level of circulating BSP was increased after tumor resection (Fig. 3a). Again, we tested whether postoperative BSP levels might also reflect the patients’ prognosis. However, neither the 50^th^ percentile cut-off value (3070 pg/ml) nor the ideal postoperative cut-off value (3608 pg/ml) that we established as described above were able to significantly discriminate between long-term survivors and patients who deceased early (Fig. 3b,c). Nevertheless, we observed a trend towards a reduced overall survival in patients with postoperative BSP serum concentrations above 3608 pg/ml (p = 0.063, Fig. 3c). Univariate Cox-regression analysis showed a similar trend for postoperative BSP serum levels above the ideal cut-off value regarding the prediction of overall survival (HR: 2.004, p = 0.070, 95% CI: 0.946–4.245). Finally, we compared the overall survival of pancreatic cancer patients who showed increasing BSP levels after surgery (n = 29) to patients with decreasing postoperative BSP levels (n = 12) to evaluate if the individual course of BSP levels over time has an impact on the patients’ prognosis. However, we observed no difference in overall survival between these two groups (Fig. 3d).

### High tumoral BSP expression is associated with a poor prognosis

**Figure 4:**
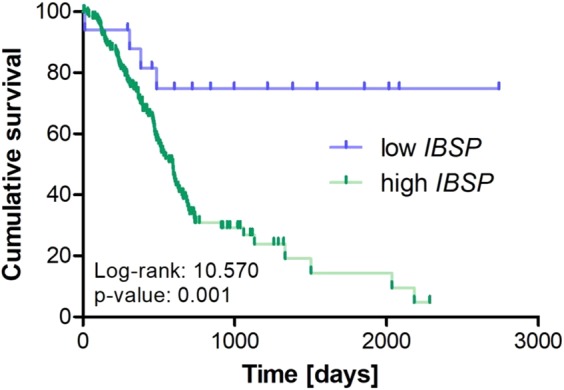
Tumoral BSP expression is associated with patients’ prognosis. Pancreatic cancer patients with high BSP mRNA expression levels in the resected tumor samples show a significantly impaired long-term survival.

Based on the promising finding on a prognostic value of preoperative BSP serum levels, we finally investigated a potential prognostic value of intra-tumoral BSP expression. Due to the non-availability of the corresponding tissue samples for the analysed serum samples, we accessed the TCGA-PAAD database and downloaded the raw BSP (*IBSP*) mRNA expression data of 178 resected PDAC tumor samples. We next compared the prognosis of patients with either high or low tumoral BSP expression. Strikingly, Kaplan-Meier curve analysis revealed a significantly impaired long-term survival for patients with BSP mRNA expression levels above the ideal prognostic cut-off value in the resected tumor samples (Fig. 4, see Patients and Methods for details), corroborating the prognostic relevance of BSP in the context of resectable pancreatic cancer.

## Discussion

In the present study, we demonstrated that serum concentrations of bone sialoprotein (BSP) are elevated in a cohort of 132 patients who underwent radical tumor resection for pancreatic cancer and that high pre-operative BSP levels represent a significant marker for an unfavourable patients´ prognosis. These data do not only argue for a novel function of circulating BSP as a serum marker in pancreatic cancer but do also support a potential relevance of BSP in the pathogenesis of pancreatic cancer, as recently suggested for other members of the SIBLINGs- family^11,14,20,21^.

Despite radical tumor resection, many patients with pancreatic cancer face early tumour recurrence and therefore display a limited prognosis^22,23^. Moreover, extended pancreatic surgery is associated with frequent clinical complications with up to 5% of all resected patients dying in the early postoperative course^4^. In this clinical context, pre-operative markers that identify patients who will particularly benefit from surgery in terms of long-term survival would be of tremendous value^24^. However, the knowledge on potential biomarkers for the identification of these patients is still limited. Based on the elevated expression of BSP in malignant pancreatic tumours as well as previous reports suggesting a direct role of BSP in the pathophysiology of pancreatic cancers^11^, we analysed serum concentrations of BSP in a large and well-defined cohort of 132 patients with pancreatic cancer that underwent tumor resection. We demonstrate for the first time that circulating levels of BSP are significantly elevated in patients with pancreatic cancer. Although the diagnostic power of serum BSP levels for the differentiation of pancreatic cancer patients and healthy controls was inferior to tumor markers such as CA19-9 and CEA, the combinational use of BSP and CA19-9 revealed an excellent discriminatory power with a diagnostic sensitivity of 83.6% and a specificity of 94.6%. These data clearly suggest that, when integrated into panels of other tumor markers (including e.g. CA-19-9), the analysis of serum BSP concentrations might represent a novel tool for the diagnosis of pancreatic cancer.

We next hypothesized that pre-operative BSP serum concentration might unravel important information on the patient’s individual postoperative course and thus provide assistance in deciding whether a patient should receive surgery or not. We therefore analysed different cut-off values for serum BSP levels to best discriminate between patients with a favourable or an unfavourable clinical outcome. In our cohort, patients with BSP concentrations above 4743 pg/ml had a significantly impaired prognosis, displaying a median overall survival of 289 days, compared to 622 days in patients with BSP concentrations below 4743 pg/ml (see Fig. 3). Notably, BSP turned out as an independent prognostic marker both in uni- and multivariate Cox-regression analyses including tumor and patient specific markers (see Table 2). Although these data suggest that serum BSP levels might be used to predict patients postoperative outcome, they do not allow any statement whether an individual patient would have benefited better from other treatment modalities. Since highly intensive pre- and postoperative systemic treatment approaches have been demonstrated to ameliorate the outcome of at least selected patients^5,25^, this missing information represents a major limitation of this study. Therefore prospective clinical trials including different treatment modalities for pancreatic cancer patients are warranted to corroborate the clinical relevance of circulating BSP in the context of this disease.

Despite the underlying pathophysiological mechanisms linking elevated serum levels of BSP and an impaired patients’ prognosis is not fully understood, our observations point towards a relevant integration of BSP into the carcinogenesis of pancreatic cancer. BSP represents one of the main structural proteins of the human bone matrix. It is synthesized by various skeletal-associated cell types such as hypertrophic chondrocytes, osteoblasts, osteocytes, and osteoclasts and constitutes for approximately 12% of human non-collagenous bone proteins. BSP represents a SIBLING family member of genetically related proteins that are encoded on chromosome 4^12^. These proteins, initially identified in bone and teeth, share many structural characteristics. It is now well established that they are overexpressed in several tumours and play a critical role at different steps of cancer development such as cancer cell adhesion, proliferation, migration, invasion, metastasis and angiogenesis^26^. Altered expression levels of BSP have been found in breast cancer as well as in metastases of non-small-cell-lung cancer. Moreover, an important role for BSP has been suggested in pancreatic cancer^11^. However, since in our cohort of patients, serum BSP levels were not correlated with tumour specific characteristics such as tumour stage or grading, it is likely that BSP rather reflects general clinical mechanism affecting the patients’ prognosis. In line with this hypothesis, correlation analyses between initial BSP levels and various laboratory markers revealed a significant correlation between BSP serum levels and parameters of inflammation and liver dysfunction. Notably, these findings are in concordance with a recent analysis, demonstrating a similar correlation in critically ill patients^25^, which also displayed an unfavourable prognosis when BSP serum levels were elevated. Our present study further revealed an increase in post-operative BSP concentrations compared to pre-operative levels, arguing for a link between postoperative inflammation and elevated BSP levels. In line with this assumption, we recently found a strong positive correlation of BSP levels and markers of bacterial infection and inflammation in critically ill or septic patients^27^. Moreover, neither postoperative BSP serum levels nor changes between pre- and postoperative BSP concentrations reflected patients long-term prognosis, leaving the exact pathophysiological mechanism how BSP impacts patients’ prognosis unclear. Moreover, further studies including multiple time points of BSP measurements after tumor resection are warranted to fully elucidate the trajectory of BSP serum levels after resection of pancreatic cancer and to evaluate a potential influence on the individual course of postoperative BSP on the patients’ survival.

Our study was limited by several points. First, we used serum samples of healthy, cancer-free blood donors as a control population. As these samples were not matched for demographic, anthropometric and socioeconomic characteristics, we cannot exclude a potential bias of these parameters on circulating BSP levels. In this line of thinking, it would also be of interest to analyse BSP serum levels in additional control populations such as patients with benign pancreatic disease or PDAC precursor lesion. Second, the analyses of tumoral BSP mRNA expression and circulating BSP levels were performed in two different cohorts of PDAC patients. Thus, our data do now allow the conclusion that elevated BSP serum levels were directly linked to an increased tumoral BSP expression. Finally, the detection-antibody of the ELISA that we used to measure circulating BSP levels has a rather unspecific immunogenic region. As BSP is known to be extensively modified post-translationally including N-and O-linked glycosylation, serine and threonine phosphorylation, tyrosine sulfation, and sialylation^28^, it is very likely that the ELISA also detected other BSP forms with a molecular mass higher than 35 kDa.

Together, the present data suggest a novel diagnostic and prognostic role of BSP in PDAC patients undergoing tumor resection. However, further clinical trials including larger, independent cohorts of PDAC patients are warranted before considering a potential implementation into clinical algorithms and to fully elucidate the pathophysiological role of BSP and other SIBLING proteins in this context.

## Supplementary information

Supplementary Mateir

## Data Availability

The datasets generated during and/or analysed during the current study are available from the corresponding author on reasonable request.

## References

[1] Carrato A (2015). A Systematic Review of the Burden of Pancreatic Cancer in Europe: Real-World Impact on Survival, Quality of Life and Costs. J. Gastrointest. Cancer.

[2] Kamisawa, T., Wood, L. D., Itoi, T. & Takaori, K. Pancreatic cancer. *Lancet* 1–13 (2016). 10.1016/S0140-6736(16)00141-0 26830752

[3] Hidalgo M (2015). Addressing the challenges of pancreatic cancer: future directions for improving outcomes. Pancreatology.

[4] Lovecek M (2016). Long-term survival after resections for pancreatic ductal adenocarcinoma. Single centre study. Biomed. Pap..

[5] Conroy, T. *et al*. Unicancer GI PRODIGE 24/CCTG PA.6 trial: A multicenter international randomized phase III trial of adjuvant mFOLFIRINOX versus gemcitabine (gem) in patients with resected pancreatic ductal adenocarcinomas. | 2018 ASCO Annual Meeting Abstracts. In *ASCO Annual Meeting* abstr LBA4001 (J Clin Oncol 36, 2018, 2018).

[6] Daamen LA (2018). Systematic review on the role of serum tumor markers in the detection of recurrent pancreatic cancer. HPB (Oxford)..

[7] von Ahrens D, Bhagat TD, Nagrath D, Maitra A, Verma A (2017). The role of stromal cancer-associated fibroblasts in pancreatic cancer. J. Hematol. Oncol..

[8] Pothula SP (2016). Key role of pancreatic stellate cells in pancreatic cancer. Cancer Lett..

[9] Nielsen MFB, Mortensen MB, Detlefsen S (2016). Key players in pancreatic cancer-stroma interaction: Cancer-associated fibroblasts, endothelial and inflammatory cells. World J. Gastroenterol..

[10] Franklin O (2015). Combining conventional and stroma-derived tumour markers in pancreatic ductal adenocarcinoma. Cancer Biomark..

[11] Kaleağasıoğlu F, Berger MR (2014). SIBLINGs and SPARC families: their emerging roles in pancreatic cancer. World J. Gastroenterol..

[12] Bellahcène A, Castronovo V, Ogbureke KUE, Fisher LW, Fedarko NS (2008). Small integrin-binding ligand N-linked glycoproteins (SIBLINGs): multifunctional proteins in cancer. Nat. Rev. Cancer.

[13] Tsai W-C (2013). The correlations of LMX1A and osteopontin expression to the clinicopathologic stages in pancreatic adenocarcinoma. Appl. Immunohistochem. Mol. Morphol. AIMM.

[14] Kayed H (2007). Effects of bone sialoprotein on pancreatic cancer cell growth, invasion and metastasis. Cancer Lett..

[15] Poruk KE (2013). Serum osteopontin and tissue inhibitor of metalloproteinase 1 as diagnostic and prognostic biomarkers for pancreatic adenocarcinoma. Pancreas.

[16] Rychlíková J (2016). Osteopontin as a discriminating marker for pancreatic cancer and chronic pancreatitis. Cancer Biomark..

[17] Budczies J (2012). Cutoff Finder: a comprehensive and straightforward Web application enabling rapid biomarker cutoff optimization. PLoS One.

[18] Loosen, S. H. *et al*. Serum levels of soluble urokinase plasminogen activator receptor (suPAR) predict outcome after resection of colorectal liver metastases. *Oncotarget* (2018). 10.18632/oncotarget.25471 PMC6007468 29930748

[19] Koch A (2011). Circulating soluble urokinase plasminogen activator receptor is stably elevated during the first week of treatment in the intensive care unit and predicts mortality in critically ill patients. Crit. Care.

[20] Zhivkova-Galunska M (2010). Osteopontin but not osteonectin favors the metastatic growth of pancreatic cancer cell lines. Cancer Biol. Ther..

[21] Delany AM (2010). Matricellular proteins osteopontin and osteonectin/SPARC in pancreatic carcinoma. Cancer Biol. Ther..

[22] Coveler AL, Herman JM, Simeone DM, Chiorean EG (2016). Localized PancreaticCancer: Multidisciplinary Management. Am. Soc. Clin. Oncol. Educ. B..

[23] Kang MJ, Jang J-Y, Kim S-W (2016). Surgical resection of pancreatic head cancer: What is the optimal extent of surgery?. Cancer Lett..

[24] Ryan DP, Hong TS, Bardeesy N (2014). Pancreatic adenocarcinoma. N. Engl. J. Med..

[25] Neoptolemos JP (2017). Comparison of adjuvant gemcitabine and capecitabine with gemcitabine monotherapy in patients with resected pancreatic cancer (ESPAC-4): a multicentre, open-label, randomised, phase 3 trial. Lancet (London, England).

[26] Lamour V, Nokin M-J, Henry A, Castronovo V, Bellahcène A (2013). [SIBLING proteins: molecular tools for tumor progression and angiogenesis]. Med. Sci. (Paris)..

[27] Luedde M (2018). Elevated serum levels of bone sialoprotein during ICU treatment predict long-term mortality in critically ill patients. Sci. Rep..

[28] Kruger TE, Miller AH, Godwin AK, Wang J (2014). Bone sialoprotein and osteopontin in bone metastasis of osteotropic cancers. Crit. Rev. Oncol. Hematol..

