# Supplementary material for: Elevated serum levels of bone sialoprotein (BSP) predict long-term mortality in patients with pancreatic adenocarcinoma
Source: Sci Rep. 2019 Feb 6;9:1489. doi: 10.1038/s41598-018-38352-2 (PMC6365503; doi:10.1038/s41598-018-38352-2)
Supplement: Supplementary file 1 — Supplementary Mateir [file 41598_2018_38352_MOESM1_ESM.pdf]

Elevated serum levels of bone sialoprotein (BSP) predict long-term mortality in patients with pancreatic adenocarcinoma

- Supplementary Material -

Sven H. Loosen[1], Pia Hoening[2], Niklas Puethe[2], Mark Luedde[3], Martina Spehlmann[3], Tom F. Ulmer[4], David V. Cardenas[1], Sanchari Roy[1], Frank Tacke[1], Christian Trautwein[1], Ulf P. Neumann[4], Tom Luedde[1,2,#], Christoph Roderburg[1,#]

[1] Department of Medicine III, University Hospital RWTH Aachen, Pauwelsstrasse 30, 52074 Aachen, Germany

[2] Division of Gastroenterology, Hepatology and Hepatobiliary Oncology, University Hospital RWTH Aachen, Pauwelsstrasse 30, 52074 Aachen, Germany

[3] Department of Internal Medicine III, University Hospital of Schleswig Holstein, Campus Kiel, Rosalind-Franklin-Str. 12, 24105 Kiel; Germany

[4] Department of Visceral and Transplantation Surgery, University Hospital RWTH Aachen, Pauwelsstrasse 30, 52074 Aachen Germany

[#] These authors share senior authorship

Supplementary Table 1. Levels of various laboratory markers

	**PDAC patients** median [range]	**Healthy controls** median [range]
BSP pre-OP [pg/ml]	1719.0 [0-8288.0]	785.6 [0-5933]
BSP post-OP [pg/ml]	3070.0 [167.5-5631.0]	-
CEA [µg/l]	2.9 [0.22-76.30]	1.3 [0.3-6.3]
CA 19-9 [U/ml]	100.6 [0.6-5637.0]	5.7 [0-44.1]
Leucocyte count [cells/nl]	7.55 [2.7-22.1]	
CRP [mg/l]	8.2 [0-237.0]	
AST [U/l]	31.5 [13.0-405.0]	30.0 [20.0-78.0]
ALT [U/l]	41.0 [1.0-651.0]	24.0 [5.0-82.0]
GGT [U/l]	124.0 [10.0-2138.0]	18.0 [8.0-98.0]
ALP [U/l]	126.5 [42.0-1574.0]	68.0 [40.0-100.0]
LDH	179.5 [113.0-317.0]	160.0 [60.0-204.0]
Bilirubin [mg/dl]	0.62 [0.2-26.20]	0.44 [0.1-1.46]
Creatinine [mg/dl]	0.85 [0.4-3.3]	
Platelets [cells/nl]	267.5 [117.0-799.0]	

BSP: bone sialoprotein, CEA: carcinoembryonic antigen, CA 19-9: carbohydrate-Antigen 19-9, CRP: C-reactive protein, AST: aspartate transaminase, ALT: alanine transaminase, GGT: γ-Glutamyl transpeptidase, ALP: alkaline phosphatase, LDH: lactate dehydrogenase

Supplementary Table 2. Correlation analysis between BSP and laboratory markers

**Parameter**	**BSP**	
	**R**	**p-value**
Sodium	-0.097	0.281
Potassium	-0.052	0.558
Leukocytes	-0.074	0.405
Thrombocytes	-0.051	0.492
AST	0.184*	0.038
LDH	0.007	0.975
Bilirubin	0.183*	0.039
ALP	0.190*	0.042
CRP	0.200*	0.033
Creatinine	-0.085	0.343

AST: aspartate transaminase, LDH: lactate dehydrogenase, ALP: alkaline phosphatase, CRP: C-reactive protein, * p<0.05

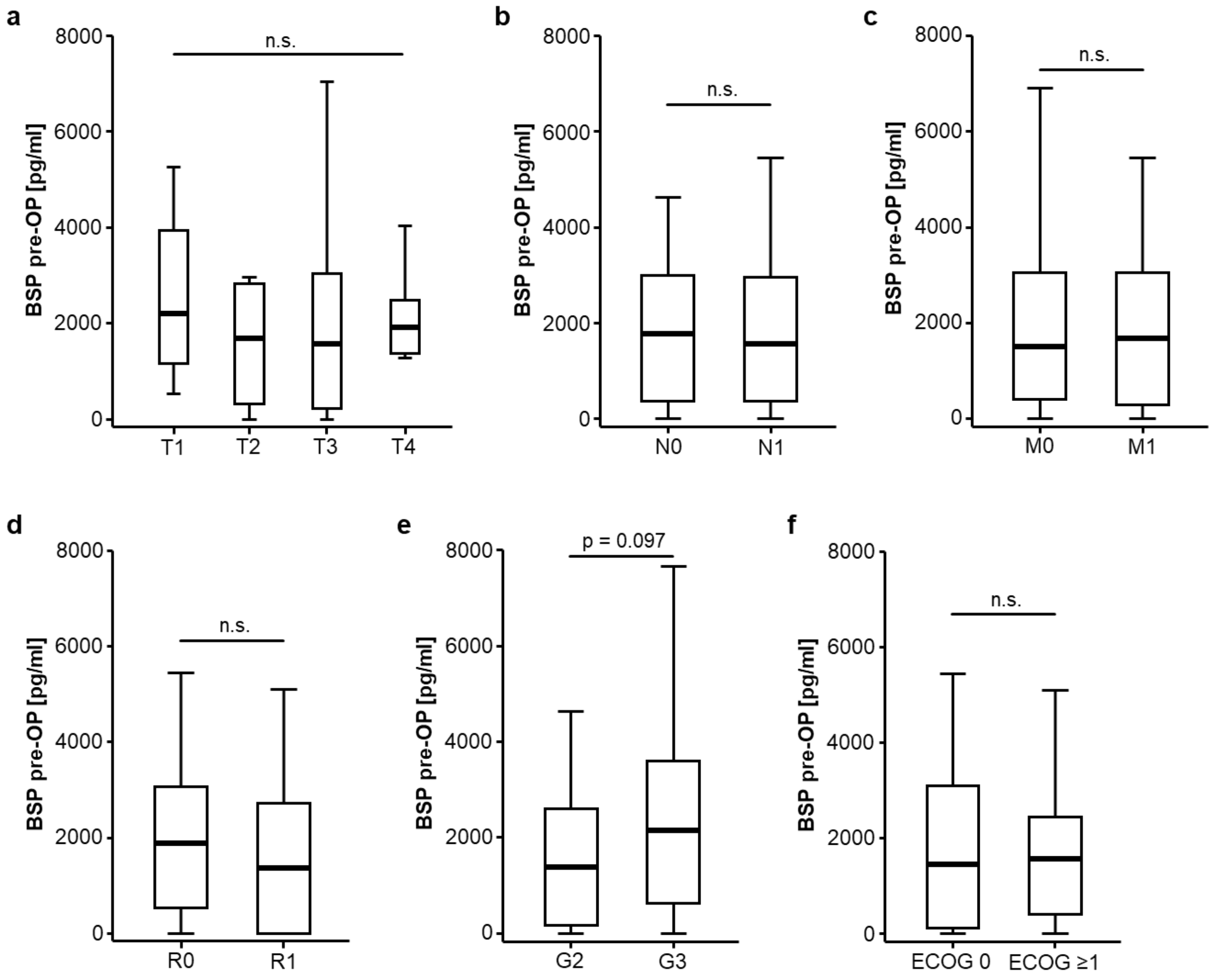

Supplementary Figure 1. Preoperative BSP serum levels do not reflect disease characteristics

BSP serum concentrations are unaltered between pancreatic cancer patients with different T-stages (a), nodal negative vs. nodal positive disease (b) as well as non-metastasized vs. metastasized patients that were still eligible for surgical tumor resection (c). BSP levels are unaltered between R0 and R1 resected patients (d). Pancreatic cancer patients with moderately differentiated tumours (G2) show a trend towards higher preoperative BSP levels compared to poorly differentiated (G3) tumours (e). BSP serum levels are unaltered between patients with an impaired/unimpaired ECOG performance status (f).
